# Convergence Deficits in Myoclonus‐Dystonia Point to Cerebellar Impairment

**DOI:** 10.1002/mdc3.70521

**Published:** 2026-01-12

**Authors:** Skadi Gerkensmeier, Christina Bolte, Jan‐Ole Radecke, Feline Hamami, Andreas Sprenger, Christoph Helmchen, Robert Chen, Marcus Callister, Talyta Cortez Grippe, Christine Klein, Norbert Brüggemann, Tobias Bäumer, Alexander Münchau, Anne Weissbach

**Affiliations:** ^1^ Department of Neurology University Hospital Schleswig‐Holstein Lübeck Germany; ^2^ Institute of Systems Motor Science, University of Lübeck Lübeck Germany; ^3^ Department of Psychiatry and Psychotherapy University of Lübeck Lübeck Germany; ^4^ Center of Brain, Behavior and Metabolism (CBBM), University of Lübeck Lübeck Germany; ^5^ Institute of Psychology II, University of Lübeck Lübeck Germany; ^6^ Krembil Research Institute, University Health Network Toronto Ontario Canada; ^7^ Division of Neurology, Department of Medicine University of Toronto Toronto Ontario Canada; ^8^ Institute of Neurogenetics, University of Lübeck Lübeck Germany; ^9^ Center for Rare Diseases, University Hospital Schleswig‐Holstein Lübeck Germany

**Keywords:** cerebellum, epsilon‐sarcoglycan, myoclonus‐dystonia, tACS, Vergence eye movements

## Abstract

**Background:**

Myoclonus‐dystonia (M‐D) is a monogenic movement disorder, with proposed cerebellar dysfunction. Vergence eye movement deficits, characteristics of degenerative cerebellar disease, have not been studied in M‐D. Cerebellar transcranial alternating current stimulation (tACS) is considered a potential therapeutic approach.

**Objectives:**

To assess vergence and prosaccade performance as markers of cerebellar dysfunction in M‐D and to evaluate the effects of cerebellar 50 Hz tACS on these eye movements.

**Methods:**

Vergence and prosaccade performance were examined in 14 M‐D patients carrying pathogenic *SGCE* variants and 14 healthy controls. A subgroup (*n* = 7) received real and sham 50 Hz cerebellar tACS in a randomized, double‐blind design.

**Results:**

M‐D patients showed prolonged latency and reduced gain of convergence compared to controls. Divergence did not differ between groups. Prosaccade peak velocity was reduced in M‐D patients. 50 Hz cerebellar tACS showed no effect on eye movements.

**Conclusion:**

Impaired convergence supports cerebellar involvement in M‐D. Further studies should identify affected pathways.

## Introduction

Myoclonus‐dystonia (M‐D) is a monogenic movement disorder, predominantly associated with a pathogenic variant in the *epsilon‐sarcoglycan* (*SGCE*) gene.^1^, ^2^ A brain‐specific isoform of the protein epsilon‐sarcoglycan is expressed in cerebellar Purkinje cells.^3^, ^4^, ^5^ The mechanism by which pathogenic variants of *SGCE* affect Purkinje cells and contribute to M‐D pathogenesis remains unclear. Purkinje cells send inhibitory output to the cerebellar nuclei via gamma‐aminobutyric acid (GABA)ergic synapses^3^ and fire at frequencies of ~50 Hz.^6^ Pathogenic variants in mouse models (eg, *Cacna1a*) that disrupt Purkinje cell function cause dystonia.^7^ An imaging study in M‐D links abnormal cerebellar microstructures with dystonia severity.^8^ Drugs that enhance GABAergic transmission, such as benzodiazepines, some anticonvulsants and alcohol, can reduce M‐D symptoms.^3^, ^9^, ^10^, ^11^ However, available treatment options are non‐specific and often ineffective.^12^

Eye movement studies in M‐D showed that saccadic adaptation as well as classical conditioning of eye‐blinks are reduced.^11^, ^13^, ^14^ Both tasks involve cerebellar‐dependent learning, arguing for a cerebellar dysfunction in M‐D. The cerebellum is crucially involved in vergence, a function that has not been studied in M‐D. Single‐unit recordings show Purkinje cell activation in dorsal vermis,^15^ posterior interposed and fastigial nuclei^16^ during vergence. Patients with cerebellar lesions including the vermis show reduced mean velocity, longer latency, and reduced gain in vergence tasks.^17^

We hypothesized that M‐D patients show impaired vergence eye movements. In a prosaccade task, we expected no differences between patients and HC, as previously reported.^13^ Furthermore, we investigated the effect of 50 Hz transcranial alternating current stimulation (tACS) of the dorsal midline cerebellum on symptom severity and oculomotor outcomes. We hypothesized that 50 Hz tACS would entrain cerebellar Purkinje cells,^18^, ^19^ thereby enhancing output to the deep cerebellar nuclei, reducing M‐D symptoms, and improving vergence performance.

## Methods

### Participants

Fourteen M‐D patients with pathogenic variants in *SGCE* (7 male, 7 female, mean age: 39.8, age range: 9–66 years) were matched with 14 healthy controls based on age (7 male, 7 female, mean age: 40.6, range: 9–68 years), handedness and sex. All patients were measured under standard medication (zonisamide in five, levodopa in two, for details, see Table S1). Patients presented with a combination of myoclonic jerks, mostly of the upper body and cervical dystonia as well as writers’ cramp. The severity of symptoms was evaluated using a video‐taped protocol. This procedure allowed a blinded rating of symptoms, reducing examination bias. We used the Unified Myoclonus Rating Scale (UMRS) as well as the Burke‐Fahn‐Marsden Dystonia Rating Scale (BFMDRS; for mean scores per measurement, see: Supplementary Table S2). Our patient's cohort showed a mean UMRS score of 24.29 (range: 1.5–88.5) and a mean BFMDRS score of 7.39 (range: 0–14.5). Patients were recruited at the University Hospital of Schleswig‐Holstein and through self‐support groups. HC reported no neurological or psychiatric disorders. The study was approved by the local ethics committee of the University of Luebeck (2023‐154_1). Written informed consent in line with the declaration of Helsinki was obtained from all participants.

### Procedure

**Figure 1:**
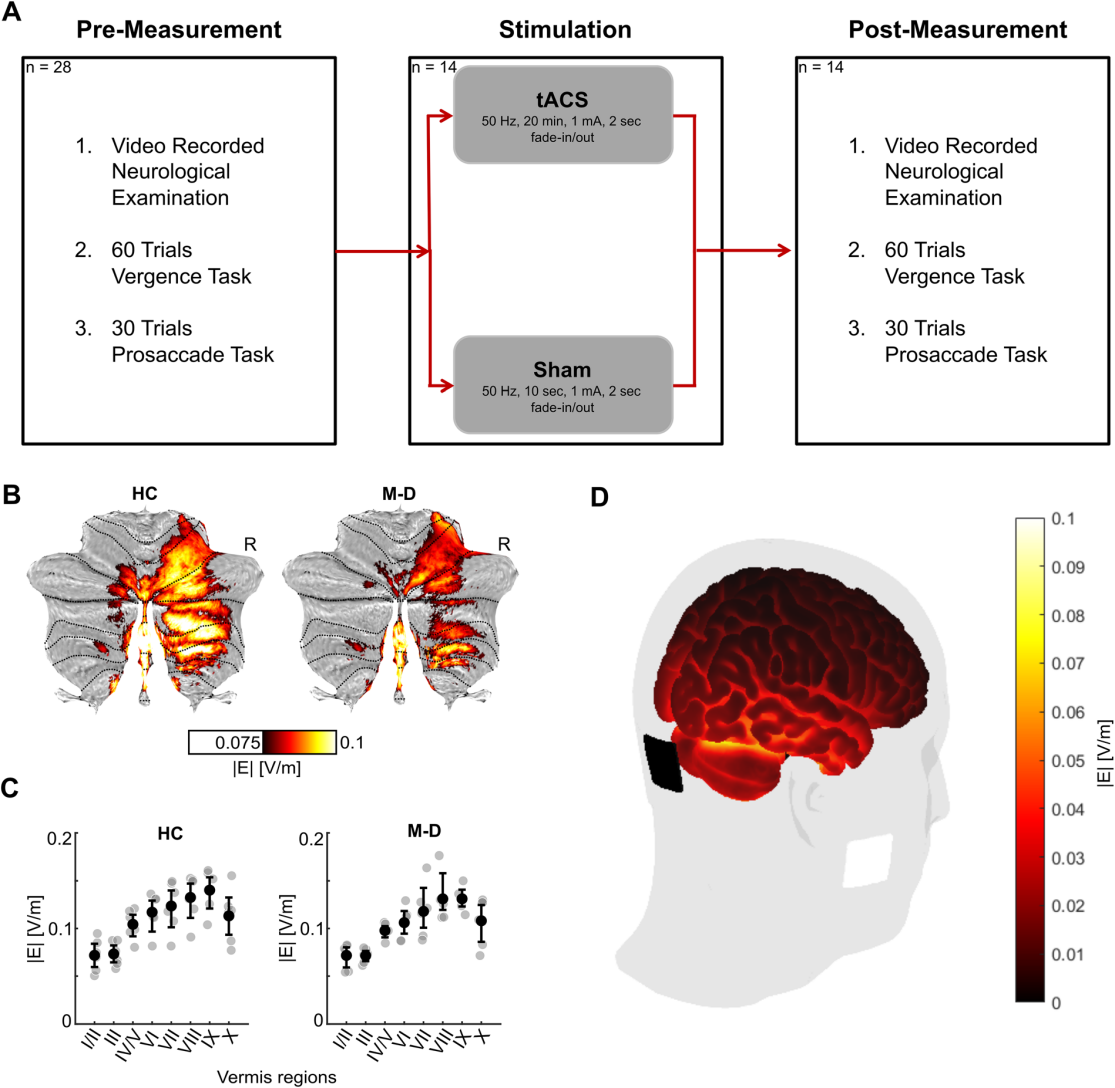
Experimental procedure and electric field simulation of cerebellar electric stimulation. (A) Participants (14 patients and 14 healthy controls) completed a baseline (pre‐measurement) session including a video‐recorded neurological examination followed by an oculomotor assessment consisting of two blocks of 30 trials of a vergence task and one block of 30 trials of a prosaccade task. A subset of participants (seven patients and seven healthy controls) underwent stimulation and the post‐measurement, resulting in two experimental days at least 1 week apart with a total of four measurements (two pre‐, two post‐measurements). In a blinded, randomized within‐subjects design, these participants received either transcranial alternating current stimulation (tACS) or sham stimulation in counterbalanced order. In the post‐measurement phase, the neurological and oculomotor examinations were repeated. The tACS condition consisted of a 50 Hz sinusoidal current applied at 1 mA for 20 min, with 2‐s fade‐in and fade‐out periods. The sham condition mimicked stimulation with identical fade‐in/out parameters, but stimulation ceased after 10 s. (B) Simulated electric field (|E|) distribution in the cerebellum for healthy controls (HC, *n* = 6) and patients with myoclonus‐dystonia (M–D, *n* = 6), highlighting strong electric field intensities in the dorsal vermis and right cerebellar cortex. (C) Electric field strength across cerebellar vermis lobules (I–X) in HC and M–D. Black dots represent group means ± standard error of the mean (SEM); gray dots indicate individual data. (D) Simulated electric field distribution on the cortical surface, indicating focal stimulation of the posterior cerebellum. Color bars denote electric field intensity (|E|, in V/m).

All 14 patients completed one session of baseline assessments of oculomotor function and symptom severity. A subset of seven patients (age range 25–66) and respective HCs received tACS. They underwent four measurements in two separate sessions, allowing for a within‐subject comparison of real and sham tACS (Fig. 1A). tACS conditions were administered in randomized order across participants. Both participants and the researcher responsible for assessing symptom severity were blinded to the stimulation condition. The researcher conducting the oculomotor assessment, however, was not blinded and administered the tACS stimulation following a standardized protocol. As the oculomotor assessment involved only minimal interaction between researcher and participant, potential bias is considered negligible. Reported side effects of stimulation did not differ between sham and real tACS.

The average delay between tACS and the post‐measurement oculomotor examination was 45 minutes, as clinical assessments were conducted first. The one‐week interval between sessions served to minimize any potential carry‐over effects. For details, see Data S1.

### Oculomotor Examination

**Figure 2:**
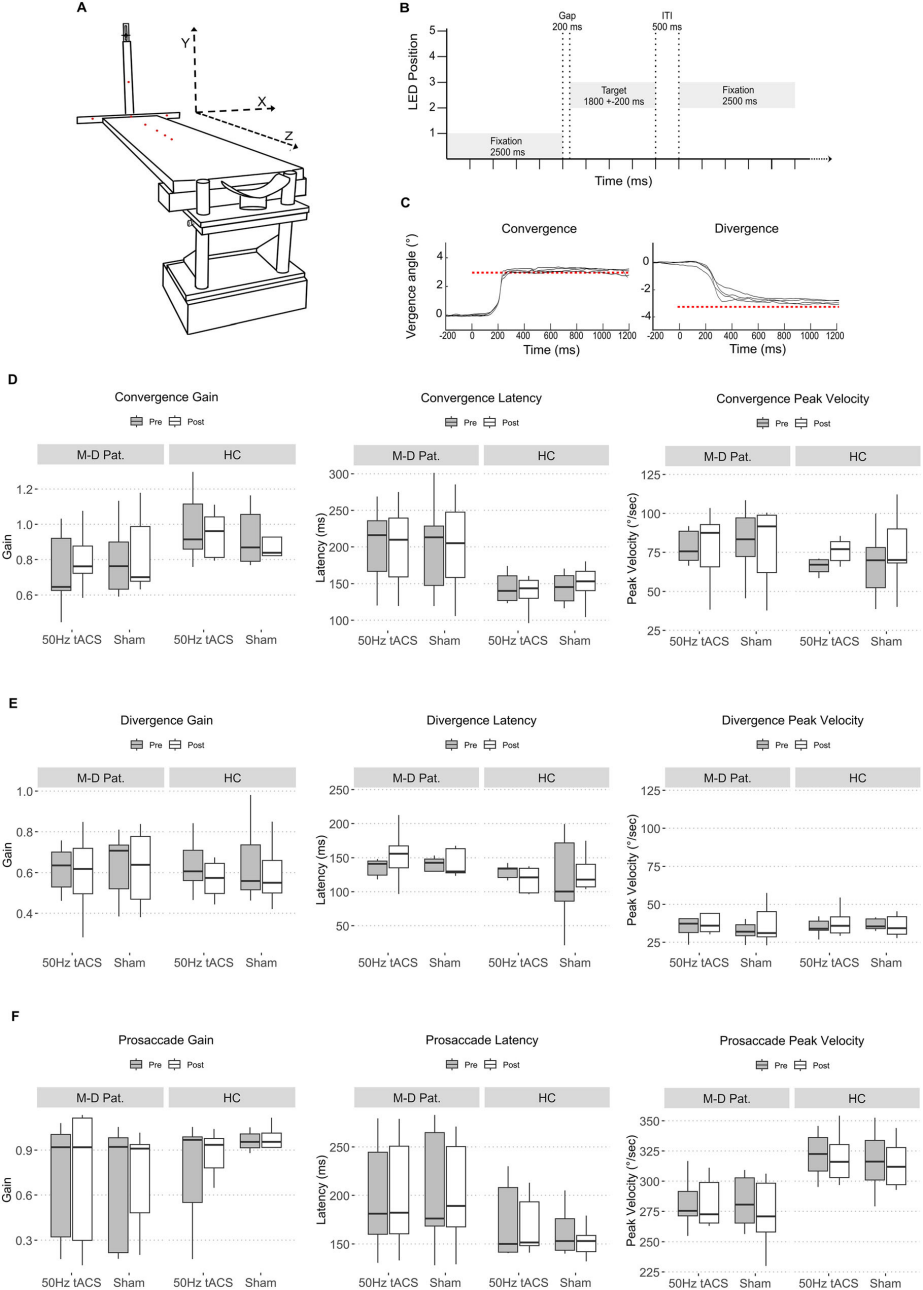
Vergence and prosaccade performance in patients with myoclonus‐dystonia (M–D) and healthy controls (HC) before (Pre) and after (Post) 50 Hz transcranial alternating current stimulation (tACS) or sham stimulation. (A) Schematic illustration of the experimental setup for measuring vergence and saccadic eye movements using an LED array in three‐dimensional space. (B) Trial timeline for the vergence tasks, showing fixation (2500 ms), a 200 ms gap, target duration (1800 ± 200 ms), and inter‐trial interval (ITI; 500 ms). (C) Eye movement traces during convergence (left) and divergence (right) for representative trials; red dashed lines indicate target vergence angle. (D–F) Boxplots depict gain, latency, and peak velocity for convergence (top row), divergence (middle row), and prosaccade (bottom row) eye movements. Data are shown separately for M–D patients and healthy controls (HC) before (Pre, gray boxes) and after (Post, white boxes) either 50 Hz cerebellar tACS or sham stimulation. Box plots represent median and interquartile ranges (IQR); whiskers extend to 1.5 × IQR. Note. Plotted values represent uncorrected descriptive data; all statistical analyses were performed on residuals regressed by age and age^2^.

Eye movements were measured with a custom‐built apparatus containing eight light‐emitting diodes (LEDs) on three axes (Fig. 2A). The arrangement of LEDs allowed measurement of step‐vergence movements with four amplitudes (1–4°) and horizontal prosaccades with 10° amplitude.^20^ Participants completed two blocks of 30 trials of the vergence task and one block of 30 trials of the prosaccade task for each measurement (for details see.: Fig. 2A–C). A chinrest was used to keep the head stable but minimize discomfort in patients with head myoclonus or cervical dystonia during the examination. The LEDs were connected to the computer via a parallel port and controlled by MATLAB (version R2022b, MathWorks, Natick/MA) using the Psychophysics Toolbox 3 extension (version 3.0.16,^21^). Binocular eye movements were recorded using the EyeLink II system (sampling rate 500 Hz, version 2.31; SR Research Ltd, Osgoode ON, Canada). Raw data was calibrated by a custom procedure (see: Data S1). Measurements were conducted in nearly complete darkness.

### tACS Protocol

Electrical stimulation followed a previously published protocol,^19^, ^22^ using a DC stimulator plus (neuroCare, Munich, Germany) in sinus mode at 1 mA with 2‐s on/off ramps. Stimulation frequency was 50 Hz, starting at 0° phase offset. Since cerebellar tACS is most effective at rest,^19^ participants were instructed to sit relaxed and avoid speaking during the 20 min of tACS. tACS electric fields were simulated based on available individual T1‐weighted MRI data (3 T Magnetom Skyra, Siemens, Germany) of six patients and respective controls (Fig. 1B–D) using MATLAB and the ROAST toolbox. For details, see Data S1.

### Statistical Analysis

The main outcome measures for the vergence and prosaccade task were peak velocity, gain (eye amplitude/target amplitude), and latency, in line with previous studies.^17^, ^23^ All outcomes were controlled for the influence of age and amplitude (see Data S1). Only trials with correct responses were included (12% [23–58%] of trials excluded in patients, 9% [17–31%] in HC). For the group comparison of baseline performance, data from the two pre‐measurements were pooled for participants who received tACS. Spearman's rho correlations were calculated between vergence outcomes and clinical measures in patients only.

All analyses were conducted in R (Version 4.4.2,^24^) using RStudio. Unless otherwise specified, the significance level was set at *α* = 0.05.

## Results

### Baseline Differences in Convergence between Patients with M‐D and Healthy Controls

An independent sample t‐test revealed that convergence latency was significantly longer in patients compared to HC (Fig. 2), *t*(26) = 2.43, *p* = 0.022. Furthermore, patients showed a reduced convergence gain compared to HC, *t*(26) = −2.44, *p* = 0.022. Convergence peak velocity showed no significant difference between patients and HC, *t*(26) = 1.62, *p* = 0.118. Divergence latency, gain or peak velocity performance was not different between patients and HC (all *p* > 0.10).

For prosaccades, we found no difference in latency *t*(25) = 0.96, *p* = 0.346 or gain *t*(25) = −0.9, *p* = 0.375, but reduced peak velocity in patients with M‐D compared to HC *t*(25) = −2.83, *p* = 0.009.

Correlation analysis revealed no significant correlations between vergence performance, and clinical scores measured by the BFMDRS and UMRS. Convergence latency was not correlated with UMRS scores (ρ = −0.21, *p* = 0.46) or BFMDRS scores (ρ = 0.07, *p* = 0.82). Similarly, convergence gain showed no significant correlation with UMRS (ρ = 0.27, *p* = 0.36) or BFMDRS scores (ρ = 0.02, *p* = 0.95).

### No Effects of 50 Hz Cerebellar tACS on Vergence Performance

Electric field simulations showed maximal stimulation intensities in dorsal vermis subregions of both groups (Vermis IX, HC: 0.14 ± 0.02 V/m, M‐D: 0.13 ± 0.01 V/m; Fig. 1B–D). To test whether 50 Hz tACS improved oculomotor performance, we calculated mixed ANOVAs with within‐subject factors STIMULATION (50 Hz tACS, Sham) and MEASURMENT (pre, post) and between‐subject factor GROUP (M‐D, HC) for latency, gain and peak velocity of convergence, divergence and prosaccades. None of the models revealed a significant main effect or interaction of STIMULATION and MEASUREMENT (all *p* > 0.05). Furthermore, there were no significant reductions of UMRS (see Table S3) or BFMDRS (Table S4) scores following stimulation.

## Discussion

Patients with M‐D showed reduced gain and longer latencies of convergence eye movements compared to healthy controls. This aligns with reports of convergence impairment in patients with spinocerebellar ataxia^25^ and cerebellar lesions.^26^ In contrast, we did not find differences in divergence performance between patients and controls. Convergence and divergence are controlled by different neural pathways: While the divergence pathway engages the ventral paraflocculus, posterior interposed nuclei and oculomotor vermis, the convergence pathway involves the oculomotor vermis and caudal fastigial nuclei.^27^ This raises the question of whether M‐D affects specific cerebellar circuits selectively. Single‐cell recordings in rhesus monkey show that the majority of dorsal vermis Purkinje cells react solely on convergence, while divergence engages stronger activation in the floccular‐posterior interposed nuclei pathway.^15^, ^28^ The isolated convergence impairment in our M‐D cohort therefore highlights the specific importance of vermal Purkinje cell dysfunction as a potential pathophysiological mechanism in M‐D.

Patients with M‐D furthermore showed reduced prosaccade peak velocity, possibly reflecting a speed–accuracy trade‐off from impaired cerebellar fine‐tuning or extracerebellar involvement.^29^, ^30^ However, given the absence of saccadic dysmetria and inconsistent prior findings,^13^ the cerebellar contribution remains uncertain.

Unlike previous examinations of eye blink conditioning or saccadic adaptation, where learning effects would overshadow treatment effects, examining vergence eye movements allows assessment of treatment effects. We compared pre‐ and post‐measurement performance between real and sham stimulation and found no differences. Electric field simulations showed that our stimulation montage effectively targeted the dorsal cerebellar vermis,^19^ and prior work has demonstrated that 50 Hz tACS can entrain Purkinje cell activity in rodents.^18^ Furthermore, a comparative study of our workgroup revealed that, compared to transcranial direct current and transcranial ransom noise stimulation, 50 Hz tACS had the strongest and longest effect on cortico‐spinal excitability.^22^ Nevertheless, some methodological constraints have to be considered: There was a 45‐minute delay between tACS and oculomotor examination, as patients’ symptoms were assessed first. While two studies of our workgroup found enhanced cortical excitability for up to an hour after cerebellar tACS,^19^, ^22^ other studies failed to find long‐lasting effects.^31^, ^32^ Furthermore, the stimulation intensity of 1 mA is relatively low. Though the electric field simulation indicates a substantial stimulation of the cerebellar vermis, other studies found effects only when using higher intensities of 1.5 mA.^33^, ^34^

In conclusion, impaired convergence with preserved divergence performance in M‐D supports the involvement of dorsal vermis Purkinje cells in M‐D pathophysiology. The convergence impairment did not correlate with symptom severity in patients.

## Authors Roles

(1) Research project: A. Conception, B. Organization, C. Execution; (2) Statistical Analysis: A. Design, B. Execution, C. Review and Critique; (3) Manuscript Preparation: A. Writing of the first draft, B. Review and Critique.

S.G.: 1A, 1B, 1C, 2A, 2B, 3A, 3B.

C.B.: 1C, 2A, 2B, 3A, 3B.

J.O.R.: 2B, 2C, 3A, 3B.

F.H.: 1A, 1B, 1C, 3B.

A.S.: 1A, 1B, 2A, 2B, 3A, 3B.

C.H.: 1A, 3A, 3B.

R.C.: 1C, 3B.

M.N.C.: 1C, 3B.

T.G.: 1C, 3B.

C.K.: 1B, 3B.

N.B.: 1B, 3B.

T.B.: 1A, 3B.

A.M.: 1A, 3B.

A.W.: 1A, 1B, 1C, 2C, 3A, 3B.

## Disclosures

**Ethical Compliance Statement:** This study was reviewed and approved by the Ethics Committee of the University of Luebeck, approval number 2023‐154_1. All procedures were conducted in accordance with the ethical standards laid down in the Declaration of Helsinki and its later amendments. Written informed consent was obtained from all participants prior to inclusion in the study and was documented in accordance with institutional requirements. We confirm that we have read the Journal's position on issues involved in ethical publication and affirm that this work is consistent with those guidelines.

**Funding Sources and Conflict of Interest:** This project was funded by the Deutsche Forschungsgemeinschaft (DFG; WE5919/2–1) and the Dystonia Medical Research Foundation (The Connie and Jim Brown early‐stage investigator award in Myoclonus Dystonia). JOR was funded by the DFG (LE 1122/7–1) and the Clinician Scientist program of the Section of Medicine of the University of Lübeck. The authors declare that there are no conflicts of interest relevant to this work.

**Financial Disclosures for the previous 12 months of all authors:** In the previous 12 months, SG received funding in the form of salaried employment at the Department of Neurology, University Hospital Schleswig‐Holstein, Lübeck, Germany, and reported no stock ownership, consultancies, advisory board participation, partnerships, honoraria, grants, intellectual property rights, expert testimony, contracts, royalties, or other financial relationships. In the previous 12 months, CB received funding in the form of salaried employment at the Institute of Systems Motor Science, University Hospital Schleswig‐Holstein, Lübeck, Germany, and reported no stock ownership, consultancies, advisory board participation, partnerships, honoraria, grants, intellectual property rights, expert testimony, contracts, royalties, or other financial relationships. In the previous 12 months, J‐OR received funding in the form of research grants from the German Research Foundation (DFG; LE 1122/7–1) and the Clinician Scientist Program of the Section of Medicine of the University of Lübeck, and salaried employment at the Department of Psychiatry and Psychotherapy, University Hospital Schleswig‐Holstein, Lübeck, Germany, and reported no stock ownership, consultancies, advisory board participation, partnerships, honoraria, intellectual property rights, expert testimony, contracts, royalties, or other financial relationships. In the previous 12 months, FH received funding in the form of salaried employment at Deutsche Rentenversicherung Bund, Reha‐Zentrum Mölln, Klinik Hellbachtal, and reported no stock ownership, consultancies, advisory board participation, partnerships, honoraria, grants, intellectual property rights, expert testimony, contracts, royalties, or other financial relationships. In the previous 12 months, AS received funding in the form of salaried employment at University Hospital Schleswig‐Holstein, Lübeck, Germany, and reported no stock ownership, consultancies, advisory board participation, partnerships, honoraria, grants, intellectual property rights, expert testimony, contracts, royalties, or other financial relationships. In the previous 12 months, CH received funding in the form of research grants from the Deutsche Forschungsgemeinschaft (HE 2689/6–1) and salaried employment at the University of Lübeck and University Hospital Schleswig‐Holstein, Lübeck, Germany, and reported no stock ownership, consultancies, advisory board participation, partnerships, honoraria, intellectual property rights, expert testimony, contracts, royalties, or other financial relationships. In the previous 12 months, RC received funding in the form of research grants from the Canadian Institutes of Health Research, the Natural Sciences and Engineering Research Council of Canada, the Parkinson Foundation, the Dystonia Medical Research Foundation, the National Organization for Rare Disorders, and AbbVie; consulting fees from AbbVie, Merz, Ipsen, and Attune Neuroscience; and reported no stock ownership, advisory board participation, partnerships, honoraria, intellectual property rights, expert testimony, employment, contracts, royalties, or other financial relationships. In the previous 12 months, MNC received funding in the form of salaried employment at the Department of Neurology, Massachusetts General Hospital, Boston, Massachusetts, USA, and reported no stock ownership, consultancies, advisory board participation, partnerships, honoraria, grants, intellectual property rights, expert testimony, contracts, royalties, or other financial relationships. In the previous 12 months, TG received funding in the form of salaried employment at the Department of Neurology, University of Toronto, and reported no stock ownership, consultancies, advisory board participation, partnerships, honoraria, grants, intellectual property rights, expert testimony, contracts, royalties, or other financial relationships. In the previous 12 months, CK received funding in the form of research grants from the Michael J. Fox Foundation, ASAP and the Deutsche Forschungsgemeinschaft; consulting fees from Centogene, Takeda, and Bial; honoraria from Bial; royalties from Oxford University Press and Springer Nature; and salaried employment at the University of Lübeck and University Hospital Schleswig‐Holstein, Lübeck, Germany, and reported no stock ownership, advisory board participation, partnerships, intellectual property rights, expert testimony, contracts, or other financial relationships. In the previous 12 months, NB received funding in the form of research grants from the Deutsche Forschungsgemeinschaft (BR4328.2–1, GRK1957), the Michael J. Fox Foundation, and the EU Joint Programme—Neurodegenerative Disease Research; honoraria from AbbVie, Esteve, Ipsen, Merz, Takeda, Teva, and Zambon; advisory board participation with Zambon; and salaried employment at the University of Lübeck and University Hospital Schleswig‐Holstein, Lübeck, Germany, and reported no stock ownership, consultancies, partnerships, intellectual property rights, expert testimony, contracts, royalties, or other financial relationships. In the previous 12 months, TB received funding in the form of research grants from the Deutsche Forschungsgemeinschaft (FOR 2698) and the Federal Ministry of Education and Research; consulting fees, advisory board fees, and honoraria from Merz Therapeutics, AbbVie, and Ipsen Pharma; contractual support from Kinderzentrum Pelzerhaken, Neustadt, Germany; and salaried employment at the University of Lübeck and University Hospital Schleswig‐Holstein, Lübeck, Germany, and reported no stock ownership, partnerships, intellectual property rights, expert testimony, royalties, or other financial relationships. In the previous 12 months, AM received funding in the form of research grants from the Deutsche Forschungsgemeinschaft (FOR 2698) and the European Reference Network for Rare Neurological Diseases; consulting fees from PTC Therapeutics; honoraria from Desitin, Teva, and Takeda; advisory board participation with the German Tourette Syndrome Association and the Alliance of Patients with Chronic Rare Diseases; royalties from Oxford University Press; commercial research support from Allergan, Ipsen, Merz Pharmaceuticals, and Actelion; and salaried employment at the University of Lübeck and University Hospital Schleswig‐Holstein, Lübeck, Germany. In the previous 12 months, AW received funding in the form of research grants from the German Research Foundation (WE5919/2–1, WE5919/4–1, and FOR2698/2), the Dystonia Medical Research Foundation, and the Michael J. Fox Foundation; salaried employment at University Hospital Schleswig‐Holstein, Lübeck, Germany; and reported no stock ownership, consultancies, advisory board participation, partnerships, honoraria, intellectual property rights, expert testimony, contracts, royalties, patents, inventions, or other financial relationships.

## Supporting information


**TABLE S1.** Clinical Characteristics and genetic variants in patients with myoclonus‐dystonia. All patients were heterozygous carriers of a pathogenic variant in SGCE. Genetic variants are described according to HGVS nomenclature (NM_003919.3 for SGCE), where c. refers to nucleotide changes in the coding sequence and p. to the resulting protein change. “Ter” denotes a premature stop codon, “fs” a frameshift, “delEx” an exon deletion, and “p.?” an uncertain protein consequence due to splicing. UMRS = Unified Myoclonus Rating Scale; BFMDRS = Burke–Fahn–Marsden Dystonia Rating Scale; tACS = transcranial alternating current stimulation. All participants were assessed while taking their standard medication. The column “tACS” refers to patients whose datasets were analyzed both pre‐ and post‐tACS (n = 7). For patients who were not included, reasons for exclusion are indicated by superscript numbers: 1 = excluded post‐hoc due to poor data quality; 2 = excluded due to unwillingness to receive tACS; 3 = excluded due to age < 18 years.
**TABLE S2.** Summary statistics for motor symptom ratings using the Unified Myoclonus Rating Scale (UMRS) and the Burke‐Fahn‐Marsden Dystonia Rating Scale (BFMDRS). Mean scores (± standard deviation (SD) of the mean) are shown for each measurement (Pre, Post) under real and sham transcranial alternating current stimulation (tACS) conditions. Lower scores indicate reduced symptom severity
**TABLE S3.** Results of repeated measures ANOVA on Unified Myoclonus Rating Scale (UMRS) scores. The table presents the main effects of transcranial alternating current stimulation (tACS) stimulation condition (real vs. sham), measurement (Pre vs. Post, and their interaction (tACS × Measurement). Degrees of freedom (df), mean square error (MSE), *F*‐values, *p*‐values, and partial eta squared (*η* ^2^) are reported. No significant main effects or interactions were observed
**TABLE S4.** Results of repeated measures ANOVA on Burke‐Fahn‐Marsden Dystonia Rating Scale (BFMDRS) scores. The table presents the main effects of transcranial alternating current stimulation (tACS) stimulation condition, measurement (Pre vs. Post), and their interaction (tACS × Measurement). Degrees of freedom (df), mean square error (MSE), *F*‐values, *p*‐values, and partial eta squared (*η* ^2^) are reported. No significant main effects or interactions were observed

## Data Availability

The data that support the findings of this study are available from the corresponding author upon reasonable request.
