# Supplementary material for: Convergence Deficits in Myoclonus‐Dystonia Point to Cerebellar Impairment
Source: Mov Disord Clin Pract. 2026 Jan 12;13(6):1488–95. doi: 10.1002/mdc3.70521 (PMC13306908; doi:10.1002/mdc3.70521)
Supplement: Supplementary file 1 — TABLE S1. Clinical Characteristics and genetic variants in patients with myoclonus‐dystonia. All patients were heterozygous carriers of a pathogenic variant in SGCE. Genetic variants are described according to HGVS nomenclature (NM_003919.3 for SGCE), where c. refers to nucleotide changes in the coding sequence and p. to the resulting protein change. “Ter” denotes a premature stop codon, “fs” a frameshift, “delEx” an exon deletion, and “p.?” an uncertain protein consequence due to splicing. UMRS = Unified Myoclonus Rating Scale; BFMDRS = Burke–Fahn–Marsden Dystonia Rating Scale; tACS = transcranial alternating current stimulation. All participants were assessed while taking their standard medication. The column “tACS” refers to patients whose datasets were analyzed both pre‐ and post‐tACS (n = 7). For patients who were not included, reasons for exclusion are indicated by superscript numbers: 1 = excluded post‐hoc due to poor data quality; 2 = excluded due to unwillingness to receive tACS; 3 = excluded due to age < 18 years. TABLE S2. Summary statistics for motor symptom ratings using the Unified Myoclonus Rating Scale (UMRS) and the Burke‐Fahn‐Marsden Dystonia Rating Scale (BFMDRS). Mean scores (± standard deviation (SD) of the mean) are shown for each measurement (Pre, Post) under real and sham transcranial alternating current stimulation (tACS) conditions. Lower scores indicate reduced symptom severity TABLE S3. Results of repeated measures ANOVA on Unified Myoclonus Rating Scale (UMRS) scores. The table presents the main effects of transcranial alternating current stimulation (tACS) stimulation condition (real vs. sham), measurement (Pre vs. Post, and their interaction (tACS × Measurement). Degrees of freedom (df), mean square error (MSE), F‐values, p‐values, and partial eta squared (η 2) are reported. No significant main effects or interactions were observed TABLE S4. Results of repeated measures ANOVA on Burke‐Fahn‐Marsden Dystonia Rating Scale (BFMDRS) scores. [file MDC3-13-1488-s001.docx]

**Supplementary Material**

Convergence Deficits in Myoclonus-Dystonia Point to Cerebellar Impairment.

**Supplementary Methods**

**Participants**

Of the 14 patients with Myoclonus-Dystonia included in our sample, at least one baseline dataset was collected from each participant. Among the 10 participants who underwent the tACS intervention, pre-tACS data from both experimental days were pooled to form the baseline dataset.
For the complete pre- and post-tACS datasets, data from three of these participants had to be excluded post-hoc due to poor data quality in the post-tACS measurements, primarily resulting from excessive blinking and/or fatigue toward the end of the experimental day. Pre-tACS measurements were repeated until data quality was sufficient, whereas post-tACS measurements were not repeated for two reasons: first, these recordings were time-critical, as they were intended to be conducted as close to the tACS intervention as possible; and second, we aimed to avoid potential confounding of treatment effects by repeating measurements until performance improved.

**Transcranial alternating current stimulation (tACS)**

Stimulation followed the protocol by Herzog et al. (1,2), using a DC stimulator plus (neuroCare, Munich, Germany) in sinus mode at 1 mA with 2-second on/off ramps. Stimulation frequency was 50 Hz, starting at 0° phase offset. Real stimulation consisted of 60,000 × 2π sinus periods (20 minutes), sham of 500 × 2π (10 seconds). Rectangular 3×3cm rubber electrodes evenly covered with Ten20 conductive paste (Weaver and Company, USA) were used yielding a current density of 0.11 mA/cm². Skin was prepared with Nuprep gel (Weaver and Company, USA) to keep impedance below 6 kΩ. Electrodes were placed neuronavigated (Brainsight neuronavigation, Rogue Research, Canada and Polaris Vicra, Northern Digital, Canada) based on acquired T1 weighted magnetic resonance images, with the anode over right cerebellar lobe VIIIa and the cathode over the right masseter muscle (‘Celnik montage’;(2,3)). tACS electric fields were simulated based on available individual T1-weighted MRI data (3T Magnetom Skyra, Siemens, Germany; 64-channel head coil, MP-RAGE sequence, 1900ms TR, 2.44ms TE, 900ms TI, 9° FA, 1mm³ resolution, 192 x 256 x 256 mm FoV) of 6 patients and respective controls using MATLAB and the ROAST toolbox (2).

**Table 1**. Clinical Characteristics and Genetic Variants in Patients with Myoclonus-Dystonia

| **ID** | **Variant in *SGCE***  **(NM_003919.3)** | **UMRS** | **BFMDRS** | **Age** | **Sex** | **Medication (daily dose)** | **tACS** |
| --- | --- | --- | --- | --- | --- | --- | --- |
| **01** | c.304C>T; p.Arg102Ter  (nonsense; pathogenic) | 19,25 | 8,25 | 41 | Female | - | No^1^ |
| **02** | c.551T>C;p.(Leu184Pro)  (missense; pathogenic) | 45,00 | 10,50 | 54 | Female | Xyrem (7 g), Zonisamid (200 mg) | No^2^ |
| **03** | c.551T>C;p.(Leu184Pro)  (missense; pathogenic) | 8,00 | 0,00 | 12 | Male | - | No^3^ |
| **04** | c.390+2T>C;p(?) (splicing variant; pathogenic) | 8,50 | 4,75 | 39 | Male | Zonisamid (200 mg) | Yes |
| **05** | c.289C>T; p.Arg97Ter  (nonsense; pathogenic) | 15,50 | 6,75 | 25 | Male | Zonisamid (200 mg) | Yes |
| **06** | c.289C>T; p.Arg97Ter  (nonsense; pathogenic) | 40,00 | 12,00 | 53 | Male | - | Yes |
| **07** | c.483delA; p.Ala162GlnfsTer8 (frameshift; pathogenic) | 88,50 | 12,75 | 66 | Female | Zonisamid (200 mg) | No^1^ |
| **08** | c.483delA; p.Ala162GInfsTer8  (frameshift; pathogenic) | 13,00 | 3,00 | 12 | Male | Levodopa (100mg/25mg) | No^3^ |
| **09** | delEx2 (large deletion,pathogenic) | 21,00 | 7,00 | 61 | Male | - | Yes |
| **10** | c.165_166insC; p.Val56ArgfsTer13  (frameshift; pathogenic) | 1,5 | 2,00 | 40 | Male | - | Yes |
| **11** | c.289C>T; p.Arg97Ter  (nonsense; pathogenic) | 24,00 | 3,50 | 9 | Female | - | No^3^ |
| **12** | c.232+1G>T; p.?  (splicing variant; pathogenic) | 12,25 | 14,50 | 59 | Female | Zonisamid (75 mg) | No^1^ |
| **13** | c.104T>A; p.Leu35Gln  (missense; likely pathogenic) | 23,50 | 7,75 | 39 | Female | Venlaflaxin, (150 mg) | Yes |
| **14** | c.104T>A; p.Leu35Gln  (missense; likely pathogenic) | 20,75 | 9,75 | 47 | Female | Levodopa (1000mg/250mg) | Yes |

**Note.** All patients were heterozygous carriers of a pathogenic variant in *SGCE*. Genetic variants are described according to HGVS nomenclature (NM_003919.3 for *SGCE*), where *c.* refers to nucleotide changes in the coding sequence and *p.* to the resulting protein change. “Ter” denotes a premature stop codon, “fs” a frameshift, “delEx” an exon deletion, and “p.?” an uncertain protein consequence due to splicing. UMRS = Unified Myoclonus Rating Scale; BFMDRS = Burke–Fahn–Marsden Dystonia Rating Scale; tACS = transcranial alternating current stimulation. All participants were assessed while taking their standard medication. The column “tACS” refers to patients whose datasets were analyzed both pre- and post-tACS (n = 7). For patients who were not included, reasons for exclusion are indicated by superscript numbers: ^1^ = excluded post-hoc due to poor data quality; ^2^ = excluded due to unwillingness to receive tACS; ^3^ = excluded due to age < 18 years.

**Oculomotor Examination**

*Eye data preprocessing*

For the custom calibration procedure, participants shifted their gaze twice from the central LED to targets along the horizontal (left/right) and vertical (up) axes (each movement covering 10° visual angle). Binocular and monocular (with one eye covered) calibrations were conducted before the pre-measurement; only binocular calibration was repeated before the post-measurement to assess calibration stability. Calibration traces were visualized, and representative samples were selected to compute the median gaze position for each target. Offset and gain values were then derived separately for horizontal and vertical orientations of each eye (4,5). Monocular calibration parameters were applied to binocular data for validation. The eye movement data was upsampled from 500 to 1000Hz using a spline interpolation (6). Two filters were applied to reduce noise: A median filter, which calculates the median of 15 samples (the data point itself and the seven data points before and after), and a Gaussian filter with a max. frequency of 30 Hz (5). The positional data of the right eye were subtracted from the positional data of the left eye, so that convergence was plotted as a positive value and divergence as a negative value (7). Vergence onset and offset were automatically detected based on eye movement velocity (5). The automatic detection scanned each trial for a peak velocity greater than 10°/sec. The beginning and end of the movement were defined by a velocity threshold of 2°/sec. This automatic, velocity-based classification was checked by visually inspecting the positional data of each trial.

For the prosaccade task, the horizontal and vertical data of the left eye was analyzed. Again, the data was automatically analyzed based on eye movement velocity. The initial velocity had to be greater than 20°/sec and the peak velocity had to be reached within 70 ms after initiation. The velocity threshold, marking the beginning and end of the saccade, was set at 20°/sec (8,9). Again, we visually checked the automatic classification based on the positional eye data. Trials in which the eyes were closed during, or shortly before the vergence or saccade, as well as trials in which no movement was initiated, were excluded from further analysis.

**Statistical Analysis**

In the vergence task, we applied a robust regression and extracted predicted values at 3° for further analysis to control for the influence of target amplitude on latency and peak velocity (10,11). For gain, the aggregated median over all amplitudes was used. In the prosaccade task, peak velocity was predicted at 10° by a robust regression. Due to the broad age range in our study, we controlled all outcomes for factor age. As we assume the effect of age to be nonlinear (12,13), we regressed latency, gain and peak velocity by age and age squared. The residuals from these models were extracted and used as the dependent variable in the further analysis.

**Supplementary Results**

**Table 2**. Summary statistics for motor symptom ratings using the Unified Myoclonus Rating Scale (UMRS) and the Burke-Fahn-Marsden Dystonia Rating Scale (BFMDRS).

| tACS | Measurement | UMRS (mean ± SD) | BFMDRS (mean ± SD) |
| --- | --- | --- | --- |
| Real | Pre | 24.8 ± 23.3 | 8.45 ± 3.67 |
| Real | Post | 20.3 ± 13.5 | 8.05 ± 3.53 |
| Sham | Pre | 26.3 ± 23.0 | 7.54 ± 5.15 |
| Sham | Post | 21.2 ± 14.0 | 8.35 ± 4.30 |

**Note:** Mean scores (± standard deviation (SD) of the mean) are shown for each measurement (Pre, Post) under real and sham transcranial alternating current stimulation (tACS) conditions. Lower scores indicate reduced symptom severity.

**Table 3.** Results of repeated measures ANOVA on Unified Myoclonus Rating Scale (UMRS) scores.

| Effect | df | MSE | F | p | η^2^ |
| --- | --- | --- | --- | --- | --- |
| tACS | 1,5 | 48.29 | 1.56 | .267 | .029 |
| Measurement | 1,5 | 11.89 | 0.04 | .844 | <.001 |
| tACS: Measurement | 1,5 | 17.99 | 1.28 | .309 | .009 |

**Note:** The table presents the main effects of transcranial alternating current stimulation (tACS) stimulation condition (real vs. sham), measurement (Pre vs. Post, and their interaction (tACS × Measurement). Degrees of freedom (df), mean square error (MSE), F-values, p-values, and partial eta squared (η²) are reported. No significant main effects or interactions were observed.

**Table 4.** Results of repeated measures ANOVA on Burke-Fahn-Marsden Dystonia Rating Scale (BFMDRS) scores.

| Effect | df | MSE | F | p | η^2^ |
| --- | --- | --- | --- | --- | --- |
| tACS | 1,6 | 4.79 | 0.15 | .711 | .002 |
| Measurement | 1,6 | 2.03 | 0.00 | .949 | <.001 |
| tACS: Measurement | 1,6 | 12.10 | 0.20 | .673 | .007 |

**Note:** The table presents the main effects of transcranial alternating current stimulation (tACS) stimulation condition, measurement (Pre vs. Post), and their interaction (tACS × Measurement). Degrees of freedom (df), mean square error (MSE), F-values, p-values, and partial eta squared (η²) are reported. No significant main effects or interactions were observed.

**References**

1. Herzog R, Berger TM, Pauly MG, Xue H, Rueckert E, Münchau A, et al. Cerebellar transcranial current stimulation – An intraindividual comparison of different techniques. Front Neurosci. 2022;16(September):1–12.

2. Herzog R, Bolte C, Radecke JO, von Möller K, Lencer R, Tzvi E, et al. Neuronavigated Cerebellar 50 Hz tACS: Attenuation of Stimulation Effects by Motor Sequence Learning. Biomedicines. 2023;11(8).

3. Celnik P. Understanding and Modulating Motor Learning with Cerebellar Stimulation. Cerebellum. 2015;14(2):171–4.

4. Rambold H, Sprenger A, Helmchen C. Effects of voluntary blinks on saccades, vergence eye movements, and saccade-vergence interactions in humans. J Neurophysiol. 2002;88(3):1220–33.

5. Sander T, Sprenger A, Neumann G, Machner B, Gottschalk S, Rambold H, et al. Vergence deficits in patients with cerebellar lesions. Brain. 2009;132(1):103–15.

6. Mack DJ, Belfanti S, Schwarz U. The effect of sampling rate and lowpass filters on saccades – A modeling approach. Behav Res Methods. 2017;49(6):2146–62.

7. Coubard O, Daunys G, Kapoula Z. Gap effects on saccade and vergence latency. Exp Brain Res. 2004;154(3):368–81.

8. Gais S, Köster S, Sprenger A, Bethke J, Heide W, Kimmig H. Sleep is required for improving reaction times after training on a procedural visuo-motor task. Neurobiol Learn Mem. 2008;90(4):610–5.

9. Sprenger A, Weber FD, Machner B, Talamo S, Scheffelmeier S, Bethke J, et al. Deprivation and recovery of sleep in succession enhances reflexive motor behavior. Cereb Cortex. 2015;25(11):4610–8.

10. Alvarez TL, Semmlow JL, Yuan W. Closely spaced, fast dynamic movements in disparity vergence. J Neurophysiol. 1998;79(1):37–44.

11. Tyler CW, Elsaid AM, Likova LT, Gill N, Nicholas SC. Analysis of human vergence dynamics. J Vis. 2012 Oct 25;12(11):21–21.

12. Rambold H, Neumann G, Sander T, Helmchen C. Age-related changes of vergence under natural viewing conditions. Neurobiol Aging. 2006 Jan;27(1):163–72.

13. Munoz DP, Broughton JR, Goldring JE, Armstrong IT. Age-related performance of human subjects on saccadic eye movement tasks. Exp Brain Res. 1998;121(4):391–400.
